# Evaluation of a community-based integrated heroin addiction treatment model in Chinese patients

**DOI:** 10.18632/oncotarget.18681

**Published:** 2017-06-27

**Authors:** Hong-He Zhang, Lin-Xiang Tan, Wei Hao, Qi-Jian Deng

**Affiliations:** ^1^ Mental Health Institute of the Second Xiangya Hospital, Central South University, The China National Clinical Research Center for Mental Health Disorders, National Technology Institute of Psychiatry, Key Laboratory of Psychiatry and Mental Health of Hunan Province, Changsha, Hunan, China; ^2^ Xiamen Xianyue Hospital, Xiamen Mental Health Center, Xiamen, Fujian, China

**Keywords:** heroin dependence, social psychological intervention, seamless connection, relapse prevention, model study

## Abstract

In this study, we analyzed the efficacy and feasibility of a community-based integrated heroin addiction treatment model in Chinese patients. The 210 heroin addicts belonging to six Chinese communities received an integrated biopsychosocial intervention that included pharmacological treatment, counseling and social assistance. High proportions of study participants were retained at the 12-month (91.9%; 193/210) and 24-month (88.1%; 185/210) follow-up visits. The number of morphine-positive subjects declined from 61.4% at baseline to 36.2% and 30.5% (*Q*=52.01; *P*<0.001) after 12 and 24 months, respectively. The crime rate decreased from 32.4% at baseline to 2.2% and 1.6% (*Q*=7.84; *P*<0.001) after 12 and 24 months, respectively. The number of patients that were employed increased from 24.3% at baseline to 37.8% and 50.8% after 12 and 24 months, respectively (*Q*=41.68; *P*<0.001). Addiction-related issues and mental health status improved according to Addiction Severity Index (ASI) and Symptom Checklist-90 (SCL-90). We therefore conclude that this community-based, integrated heroin addiction treatment model is highly feasible with high treatment retention, reduced drug use, a lower crime rate, improved health and increased employment.

## INTRODUCTION

Illicit drug abuse is a serious public health problem in China since the 1990s [1–3]. More than 2.95 million drug abusers were registered in the Public Security System of China by 2014 and heroin was the most commonly used illicit drug in China. Although heroin use has decreased since the launch of the methadone maintenance program (MMT) in 2004, it still accounts for 49.3% of registered users [4]. Drug abuse not only results in health and psychological issues to the individual, but also causes public health and social problems including crime and the HIV epidemic [5, 6].

The Chinese government introduced the Compulsory Drug Rehabilitation Program (CDRP) in the 1990s with more than 500 detoxification centers to help drug abusers undergo detoxification under the supervision of the public security or judicial departments. However, high relapse rates pose a significant challenge for the CDRP treatment. This led to the introduction of the Methadone Maintenance Treatment (MMT) program in 2004 to help heroin addicts gain easy access to continuous treatment [7]. According to the annual report on drug control in China in 2014, there were 763 MMT clinics across the nation by the end of 2013. The MMT program has been successful in preventing relapse and improving social issues [8–10]. However, social discrimination and stigma often hinder drug abusers from returning to society and living ordinary lives. Thus, apart from pharmacological therapy, patients require psycho-social interventions to ensure full rehabilitation and social reintegration.

In the late 1980s, Yunnan province introduced a therapeutic community program by establishing hierarchically arranged communities and provided specific treatments according to therapeutic community principles [11]. Changsha and Shanghai expanded this program by recruiting government social workers to help drug addicts succeed in re-entering society through psycho-social interventions such as relapse prevention programs, in-clinic counseling, and motivational interviewing [12].

Although the drug rehabilitation treatment has been successful over the past two decades with substantial support from the Chinese government, social barriers still remain. Most Chinese consider drug abuse as a bad habit, a personality flaw or moral degeneration as opposed to a chronic relapsing brain disease [13]. Moreover, they believe that drug abusers deserve punishment for their social behavior [14]. In addition, major efforts were focused on medication treatment for detoxification whereas community based psycho-social rehabilitation and relapse prevention programs were ignored. The lack of coordination among government departments in drug control and treatment contributed to high risk of relapse [15]. The health department focused on drug detoxification treatment, whereas the public security department mainly focused on catching and punishing the drug user. The justice department that assists psycho-social rehabilitation lacked expertise and had poor connection with communities.

**Figure 1:**
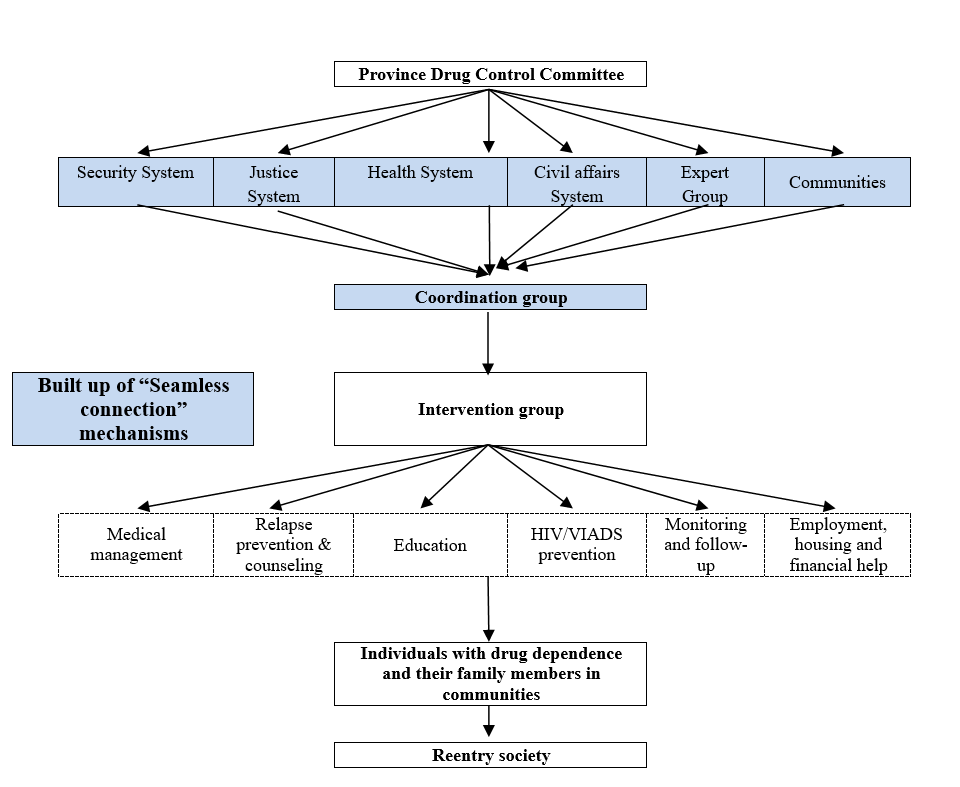
Diagram of the structure and functioning of the community-based integrated heroin dependence treatment model

Hence, ex-drug users face social stigma, discrimination, unemployment, and lack of psychosocial support when they are discharged from compulsory or voluntary treatment facilities [16]. To deal with these challenges and difficulties, we worked with government control authorities in Hunan, Shanghai and Yunnan to assess the problems associated with drug abuse, treatment and the drug control policy. By relocating drug treatment resources to communities and integrating all government and social resources, we intended to form a comprehensive mechanism for drug addiction treatment that would provide a cost-effective and systematic service, not only for drug addicts but also for their families. The treatment model is shown in Figure 1. The procedure and feedback for this community-based, integrated biopsychosocial heroin dependence treatment model has previously been published [17]. We hypothesized that this comprehensive model would reduce relapse rates and criminal activity, increase employment and assist their rehabilitation and society re-entry. Therefore, in this study, we evaluated the outcomes of implementing this model.

## RESULTS

### Baseline demographics of study subjects

Among the 210 participants in this study, 73.8% were males. The average age of the participants was 39.5 ± 7.13 years. The education level was middle school or below in most cases. Nearly 59% of the participants were single, separated or divorced and 3.3% used other substances apart from heroin. The average period of heroin use was 5.29 ± 2.88 years. These demographics were comparable among participants in all the three sites.

### Main outcome measures during follow-up

The proportion of subjects that tested positive for morphine in the urine test declined from baseline (129 /210, 61.4%,) to 12-month visit (76/210, 36.2%) and 24-month visit (64/210, 30.5%, *Q* = 52.01, *P* < 0.001; Table 1). The crime rate also reduced at the 12-month (4/185, 2.2%) and 24-month (3/185, 1.6%) visits compared to the baseline (60/185, 32.4%, *Q* = 101.37, *P* < 0.001). The employment rate increased at the 12-month (70/185, 37.8%) and 24-month (94/185, 50.8%) visits compared to baseline (45/210, 24.3%, *Q* = 41.6834.57, *P* < 0.001). Notably, the employment rate was higher at the 24-month visit compared to 12-month visit (*Q* = 7.48; *P* = 0.006).

**Table 1:** Main outcome measures at baseline and 12- and 24-month follow-up visits

Main Outcome Measures	Baseline (*n*= 210)	12-month (*n*= 193)	24 month (*n*= 185)	*Q*	*P*
Proportion of positive urine tests (*n*=210)	129(61.4%)	76(36.2%)	64(30.5%)	52.01	<0.001
Crime rate (*n*=185)	60(32.4%)	4(2.2%)	3(1.6%)	101.37	<0.001
Employment rate (*n*=185)	45(24.3%)	70(37.8%)	94(50.8%)	41.68	<0.001

*Q* refers to the statistic value obtained with Cochran's Q test.

### Analysis of addiction severity index (ASI) and symptom checklist-90 (SCL-90)

At baseline, the 210 participants showed severe occupational, physical health, family relationship, alcohol use and mental health problems (Table 2). Except for drug use, all other areas showed significant improvements at both 12- and 24-month follow-up visits. Medical status, alcohol use, occupational functioning, legal status, family/social status and psychiatric status showed considerable improvement at the 24- month follow-up compared to the 12 month follow-up. Also, we observed reduced total and subscale SCL-90 scores at 12 (*P* < 0.05) and 24 (*P* < 0.01) months compared to baseline (Table 2).

**Table 2:** The ASI subscales and SCL-90 scores at baseline and12- and 24-month follow-up visits

		Baseline (*n*=210)	12-month (*n*=193)	24 month (*n*=185)	*F*	*P*
ASI subscales	Medical status	0.29±0.43	0.21±0.30	0.17±0.28	5.92	0.003
	Alcohol use	0.20±0.28	0.12±0.17	0.08±0.12	18.36	<0.001
	Occupational functioning	0.79±0.27	0.74±0.29	0.64±0.32	13.42	<0.001
	Drug use	0.06±0.10	0.05±0.10	0.04±0.18	1.31	0.270
	Legal status	0.07±0.18	0.03±0.08	0.01±0.05	13.23	<0.001
	Family/social status	0.27±0.33	0.14±0.15	0.12±0.15	24.45	<0.001
	Psychiatric status	0.15±0.20	0.10±0.15	0.07±0.16	12.07	<0.001
SCL-90 scores	Somatization	2.90±0.68	2.66±1.28	2.55±0.64	6.80	0.001
	Obsessive-compulsive	2.88±0.67	2.65±1.31	2.53±0.56	6.86	0.001
	Interpersonal sensitivity	2.82±0.68	2.58±1.31	2.44±0.57	7.65	0.001
	depression	2.87±0.66	2.65±1.23	2.55±0.65	6.32	0.002
	Anxiety	2.79±0.67	2.55±1.30	2.41±0.54	7.99	<0.001
	Hostility	2.86±0.76	2.59±1.30	2.44±0.56	9.77	<0.001
	Phobic anxiety	2.66±0.72	2.34±1.28	2.26±0.46	10.51	<0.001
	Paranoid ideation	2.78±0.70	2.50±1.27	2.42±0.52	8.39	<0.001
	Psychoticism	2.70±0.63	2.46±1.28	2.33±0.47	8.22	<0.001
	Others	2.90±0.66	2.68±1.30	2.61±0.66	5.15	0.006
	Total	28.05±6.22	25.65±12.53	24.23±5.06	8.24	<0.001

*F* refers to statistic value obtained with one-way ANOVA.

### Demographic characteristics and drug use are not associated with treatment retention or drop-out

Table 3 summarizes the one-way ANOVA results that showed no statistical differences in demographic characteristics and drug use history of the participants who were retained in the study in comparison to those who dropped out at 12- and 24-month follow-up visits. There were no statistically significant differences detected in demographic characteristics or drug use history among the three groups.

**Table 3:** Comparison of demographic characteristics and drug use history of study subjects retained or dropped out at the 12- and 24-month visits

Characteristics	Retained at 24 month visit (*n*=185)	Drop-out at 12 month visit (*n*=17)	Drop-out at 24 month visit (*n*=8)	Statistic Value	*P*
Age (years) (mean±SD)	39.2±7.0	41.3±6.2	39.4±6.0	*F*=0.71	0.493
Years of heroin use (mean±SD)	5.6±3.0	5.0±2.0	6.4±1.8	*F*=0.61	0.547
Female (%)	50(27.0%)	4(23.5%)	1(12.5 %)	*χ*^2^=0.91	0.636
Education (%)				*χ*^2^=0.74	0.690
6 years of education	16(8.6%)	1(5.9%)	0(0.0%)	--	--
9 years of education	118(63.8%)	11(64.7%)	5(62.5%)	--	--
≥12 years of education	51(27.6%)	5(29.4%)	3(37.5%)	--	--
Unmarried (%)	105(56.7%)	11(64.7%)	7(87.5%)	*χ*^2^=3.27	0.195

*χ*^2^ refers to statistic value obtained with the Chi-square test.

Logistic regression analysis (Table 4) showed that years of drug use (Wald *χ*^2^ = 0.21, *P* = 0.648), age (Wald *χ*^2^ = 1.80, *p* = 0.180), gender (Wald *χ*^2^ = 0.34, *P* = 0.558), education (Wald *χ*^2^ = 0.77, *P* = 0.681) and marital status (Wald *χ*^2^ = 1.88, *P* = 0.171) were not associated with treatment retention or dropout.

**Table 4:** Logistic regression analysis (enter method) of possible factors of treatment dropout

Characteristics	*β*	*SE*	Wald*χ*^2^	*P*	EXP(*β*)	95%CI	
						Down	Upper
Age (years)	-0.04	0.03	1.80	0.180	0.96	0.90	1.02
Years of heroin use	0.04	0.08	0.21	0.648	1.04	0.89	1.21
Female	0.32	0.54	0.34	0.558	1.37	0.48	3.94
Education							
6 years	1.00	1.00	0.77	0.681	1.00	1.00	1.00
9 years	-0.94	1.09	0.74	0.388	0.39	0.05	3.31
≥12	-0.95	1.12	0.72	0.396	0.39	0.04	3.45
Unmarried	-0.66	0.48	1.88	0.171	0.52	0.20	1.33

*β* is a regression coefficients. *SE* is the Standard Error. Wald*χ*^2^ refers to Wald statistics. EXP(*β*) is the occurrence ratio. 95%CI is the 95% confidence interval for EXP(*β*).

## DISCUSSION

The drug addiction treatment is influenced by social, cultural, economic and ethnic factors as well as government policy and social values. All these factors are essential for successful integration of addicts into society after successful treatment. Although China has shown tremendous progress in heroin treatment, major obstacles remain. These include non-integration of compulsive and voluntary treatments, inefficient utilization of available government and community resources and lack of an integrated mechanism of drug treatment resources. Therefore, developing a comprehensive model for drug treatment is critical to improve the drug addiction treatment outcomes [15].

Government plays an important role in heroin addiction treatment by reviewing treatment demands and barriers and providing accessible and comprehensive services [25, 26]. Moreover, resources such as the relevant government departments, social workers, community leaders, and family members also need to be integrated into the treatment model [17]. In this study, relevant government departments joined the research team and worked in coordination to find solutions to the problems and barriers during the study. This resulted in a positive feedback system that was integrated both functionally and structurally (see Figure 1). Therefore, problems were identified, discussed and resolved in a timely manner. This allowed effective treatment to the heroin addicts.

Drug addicts face many social and psychological problems [25]. Therefore, effective treatment should combine pharmacological treatment with other services that meet the complex needs of the drug addicts [29]. Many studies indicate that an integrated community based treatment that includes pharmacological treatment, relapse prevention, and social assistance enhances recovery and prevents relapse [26–28].

In our study, we combined pharmacological treatment (including acute detoxification and MMT) with psychological therapies (such as psychological counseling, crisis intervention) and social assistance as part of a community-based integrated treatment model. After a 2-year follow-up, we observed high treatment retention, reduced drug use, lower crime rate, improved health and increased employment in the subjects of the study. However, we also found that drug use history, age, gender and marriage status were not related to treatment dropout, which may be attributed to the small sample size [29]. This treatment model faced challenges such as lack of finances and qualified social workers and community staff. Therefore, the local economy and social culture needs to be considered to create a more sustainable model in future studies.

A major limitation in the study was the lack of a control group. We discussed having a control group during the study design, but it was impossible to have a comparable control group in the cohort study. We viewed drug addiction as a chronic and relapse brain disorder that required appropriate intervention. Therefore, we preferred the pre- and post-intervention study design to gain insights into the efficacy of integrated community-based heroin addiction treatment in southern China. The retention rates in our study were significantly higher both at 12-month and 24-month visits compared to a 5-year retrospective multi-center cohort study of MMT at 8 community-based clinics in China (91.9% *versus* 73.1%, 2 = 34.09, *P* < 0.001; 88.1% *versus* 62.0%, *χ*^2^ = 53.53, *P* < 0.001, respectively) [30, 31]. We postulate that retention rate is more convincing than urine tests to determine intervention efficacy because urine tests reflect drug use within the previous few days and do not indicate if drug addicts have quit drug-taking.

In summary, we showed that the community-based, integrated bio-psycho-social heroin addiction treatment model was feasible and promising with high treatment retention, reduced drug use, lower crime rate, improved health and increased employment. This treatment model provided all-round accessible services to effectively promote full rehabilitation. Community-based integrated treatment in southern China still faces challenges including lack of finances and qualified social workers and community staff. Therefore, in future studies the local economy and culture needs to be considered.

## MATERIALS AND METHODS

### Ethics statement

This study was approved by the Institutional Review Boards (IRBs) and the Human Subjects Protection Committees at the Second Xiangya Hospital, Shanghai Mental Health Center, and Yunnan Institute of Drug Abuse.

### Participants

This study was carried out in six community treatment centers (two from Hunan, three from Shanghai, and one from Yunnan) from April 2008 to October 2010. Inclusion criteria for study participants were: (1) 18 years of age or older; (2) met the DSM-IV-TR diagnostic criteria for heroin addiction [18]; (3) 6 or more years of education, and (4) no other severe mental disorders. The participants were excluded if they refused oral or written informed consent and/or had serious mental and physical illness.

After screening 246 participants in the six community treatment centers, 36 were excluded from the study (11 had serious physical illness, 16 did not meet inclusion criteria, 4 refused to participate and 5 because of other causes). Finally, 210 individuals were recruited for the study, of which 90 were from Shanghai city and 60 each from Hunan and Yunnan provinces. Among these, 193 (91.9%) and 185 (88.1%) completed the 12-and 24-month follow up interviews, respectively. All participants signed informed consent forms prior to participation in the study and received 50 Yuan at each assessment point as compensation for time and inconvenience.

### Heroin addiction treatment model

In brief, coordination groups were formed at each site that included leaders from the city police security system, justice system, health system and civil affairs system as well as the Provincial Narcotics Control Committee (PNCC) and the principal investigators. The coordination group designed and implemented an optimized community based rehabilitation system for heroin addicts. The intervention group at each treatment site comprised of psychiatrists, psychologists and social workers. Community staff and policemen working in the community were also involved in organizing social support. Regular project coordination meetings were organized to discuss progress and respond to barriers through a process of rational resource allocation by the various government departments. The structural and functional relationships of the different government departments in the integrated treatment model are described in Figure 1.

The community-based intervention consisted of pharmacological treatment, counseling and social assistance. The pharmacological treatment included both acute detoxification and methadone maintenance treatment. The counseling consisted of CBT based psychological counseling, crisis intervention and HIV/AIDS prevention. The psychological counseling was largely derived from CBT based relapse prevention and motivational interviewing principles with 18 relapse prevention topics [19–21]. The PI assistants also assessed, documented, and monitored drug use. Social assistance primarily included financial support, housing and employment assistance (see Figure 1).

An experienced supervisor was responsible for training and supervision at each study site to ensure reliability and fidelity of practice. Each supervisor had worked in the addiction field for more than 10 years and was qualified in psychological counseling. Staff members in the community treatment facilities included social workers trained in psychological background and were clinically supervised.

Each study participant developed a one-year rehabilitation plan with the help of their social worker. During the first 3 months, each participant attended individual counseling every week. Then, the social workers regularly contacted the participant either in person or by phone to assess their situation. In addition, 2-hour group counseling sessions were scheduled each month. Participants were invited to attend and were given small gifts as an incentive for their participation.

To create a supportive environment, 150 family members of the study participants and local community leaders were involved in the program. Monthly educational sessions on drug addiction were offered at the local community treatment center. Related brochures were also distributed to all study participants and their family members. Additionally, a community education campaign was launched including billboard posters, flyers, and letters to local residents. These activities were aimed at improving community understanding of drug addiction and its related risk behaviors as well as diminishing negative attitudes towards drug addicts. Moreover, communities provided necessary help to drug users and their families in their daily life such as minimum allowance, shelter, job and so on.

### Study measures

After providing informed consent, participants completed questionnaires and undertook a physical examination. Study measures included the following:

(1) The Addiction Severity Index (ASI) covered medical, employment/support, drug and alcohol use, legal, family/social, and psychiatric problems. It obtained lifetime information as well as problems within the previous 30 days [21].

(2) Structured Clinical Interview for DSM-VI-TR Axis I disorders patient edition (SCID-I/P) was used to diagnose heroin dependence and other psychiatric conditions at baseline [22].

(3) The Symptom Checklist-90 (SCL-90) includes a self-report questionnaire measuring symptom severity of psychopathology in different patient populations [23]. It has subscales of somatization, obsessive-compulsive, interpersonal sensitivity, depression, anxiety, hostility, phobic anxiety, paranoid ideation, psychoticism and others. The Chinese version is used extensively in mental health with good reliability and validity [24].

(4) Urine drug screens test: Urine samples were collected at baseline, 12 month follow-up and 24 month follow-up visit as well as random tests on-site during the intervention. Drug Diagnostic Kit (Colloidal Gold, Shanghai Chemtron Biotech Inc.) was used for the urine drug test to test for morphine.

### Statistical analysis

All analyses were performed using SPSS software package (Version15.0). Chi-square test and one-way ANOVA were used for categorical and continuous variables, respectively to compare demographic characteristics, drug use history and mental health status. *Cochran's Q* test was used to analyze changes in the proportion of positive urine drug tests, criminal activity and employment.

Urine drug tests results for drop-outs and those who refused to be tested were imputed as positive. Criminal activity and employment was only analyzed for participants who were retained in the study for 24-months. Logistic regression (enter method) was used to identify possible factors associated with treatment dropout. All analyses were two-tailed comparisons. For comparison between three groups, *P* < 0.05 was considered significant, whereas *P* < 0.0167 (corrected) was considered significant for pairwise comparisons.
